# Effect of Se-Enriched Irrigation Water on the Biomass Production and Elemental Composition of Green Bean, Cabbage, Potato and Tomato

**DOI:** 10.3390/plants10102086

**Published:** 2021-10-01

**Authors:** Péter Ragályi, Tünde Takács, Anna Füzy, Nikolett Uzinger, Péter Dobosy, Gyula Záray, Nóra Szűcs-Vásárhelyi, Márk Rékási

**Affiliations:** 1 Institute for Soil Sciences, Centre for Agricultural Research, Herman O. út 15., H-1022 Budapest, Hungary; ragalyi.peter@atk.hu (P.R.); uzinger.nikolett@atk.hu (N.U.); szucs-vasarhelyi.nora@atk.hu (N.S.-V.); rekasi.mark@atk.hu (M.R.); 2 Institute of Aquatic Ecology, Centre for Ecological Research, Karolina út 29-31, H-1113 Budapest, Hungary; dobosy.peter@ecolres.hu (P.D.); zaray.gyula@ecolres.hu (G.Z.)

**Keywords:** irrigation, biofortification, selenium, recommended dietary allowance, element content

## Abstract

Additional Selenium (Se) intake may be recommended in areas of Se deficiency to prevent various human diseases. One possibility for this is biofortification. In this experiment, the effect of irrigation water containing 100 and 500 µg L^−1^ Se, in the form of Na_2_SeO_4_, on green bean, cabbage, potato and tomato was investigated in a greenhouse pot experiment with sand, silty sand and silt soils. The chlorophyll content index was usually improved by Se and was significantly higher in potato in sand and silty sand and in tomato in silty sand and silt soils. The Se content of edible plant parts increased 63-fold in the 100 µg L^−1^ Se treatment and almost 400-fold in the 500 µg L^−1^ Se treatment, averaged over the four species and the three soils. Irrigation water with a Se content of 100 µg L^−1^ may be suitable for the production of functional food in the case of green beans, potatoes and tomatoes. However, due to its greater Se accumulation, cabbage should only be irrigated with a lower Se concentration. The use of Se-enriched irrigation water might be a suitable method for Se biofortification without a significant reduction in plant biomass production and without a remarkable modification of other macro- and microelement contents.

## 1. Introduction

The selenium (Se) content of soils varies greatly worldwide, from very low to toxic levels even within the same country [1,2]. Its total concentration in the soil is relatively low, generally between 0.01 and 2 mg kg^−1^ [3], with a global average of 0.4 mg kg^−1^ [4].

However, in addition to the total Se content of the soil, the availability of Se to plants is also an important aspect which is greatly influenced by the chemical form of the element.

The presence and distribution of different Se forms in soil is a function of the interaction between soil factors. The more mobile selenate (Se^6+^) occurs primarily in alkaline and well-aerated soils, whereas selenite (Se^4+^) is less mobile and more common in neutral or acidic soils and under less oxic conditions. Selenide (Se^2-^) is relatively immobile, is formed under acidic conditions and is strongly bound to mineral or organic compounds. Under reducing conditions, selenium forms may also undergo precipitation [5]. The solubility and plant availability of Se increase with increasing soil pH [6,7]. Selenite sorption correlates well with the Fe and Al contents, so Fe oxides may also play a significant role as Se sorption sites [8]. However, Coppin et al. [9] found that in a grassland soil, Se was bound largely to the organic fraction, with only a minor part bound to mineral components.

The essential role of Se has not been proven for plants, but in certain circumstances, it may have beneficial effects [10], such as mitigating the negative effect of certain abiotic stress factors like heavy metals, drought, salinity and temperature [11,12,13]. The application of Se in certain doses may result in yield increases [14]. A low concentration of supplementary selenium in the growth medium may improve physiological processes, like the efficiency of photosynthesis [12,15,16,17], especially under abiotic stress conditions. Based on their ability to absorb Se, plant species can be classified as hyperaccumulators (>1000 mg kg^−1^ Se), secondary-accumulators (100–1000 mg kg^−1^ Se) and non-accumulators (<100 mg kg^−1^ Se) [15].

The Se concentration of the soil in a specific area determines the Se content of wild and cultivated plants and is thus closely related to the daily Se intake of the people and animals living in that area. The ability of plants to absorb Se is essential for human health since Se is known to be an essential nutrient for humans and animals. It plays a significant role as an antioxidant and has diverse effects on health [18]. In areas with a low Se supply, additional Se should be provided [19]. A more direct way to compensate for low Se intake in humans is to produce Se-enriched functional foods, a process known as biofortification [20]. In Finland, the low dietary intake of Se was improved from the 1980s onwards by mixing Na-selenate (Na_2_SeO_4_) with fertilizer [21]. Effective measures have also been taken in the UK to increase the Se content of foods [22]. In Hungary, both the Se supply in soils and the Se concentration in the blood of the population are low, so biofortification with Se should be a priority [23].

The enrichment of irrigation water, i.e., fertigation, is one way of producing functional foods in order to enhance the intake of certain elements [24,25].

The biofortification of various plant species with Se has been extensively studied [20,26,27,28]. Tomato, potato and cabbage have been given considerable attention in this respect, while less research has been reported about beans, although this is the most important legume crop for direct human consumption [29]. According to the literature, Se has been investigated in roughly equal proportions in hydroponic experiments and in a soil medium (either in the field or in pots). When soil was used as the growth medium, Se was typically applied in one or several doses, either by adding a solution to the soil or in the form of leaf spraying [30]. Selenium fertigation, usually applied to the soil throughout the whole vegetation period, has so far only been investigated to a limited extent for melon, tomato [24,25,31], broccoli and canola [32]. These studies were largely conducted in the San Joaquin Valley, California, where it is the high Se content of the irrigation water that is causing the problem [33].

However, Se fortification may have contradictory effects on the element composition of Se-treated plants. The antagonistic or synergistic effect of Se has been studied primarily in human or animal nutrition, with far less research on plant nutrition. Antagonism has been detected between Se and sulphur [34], Se and mercury [35] and Se and molybdenum [36]. The relationship of Se with arsenic species [37,38] and cadmium [39,40] was also studied and found to be either synergistic or antagonistic.

The interaction between Se and other elements may also depend on whether the studies were performed in soil or in hydroponic cultures. Feng et al. [41] investigated Chinese brake fern (*Pteris vittata*) in both hydroponic and soil culture. In plants grown in soil, Se suppressed the uptake of most of the elements tested, including magnesium (Mg), potassium (K), phosphorus (P), iron (Fe), copper (Cu) and zinc (Zn). In nutrient solution, the uptake of most essential elements was suppressed at lower levels of Se, but higher Se doses had a synergistic effect and stimulated the uptake of Ca, Mg and K. This shows that only the results obtained in soil may be relevant if we want to investigate the expected effect of Se in the field.

To the best of our knowledge, this is the first experiment to use Se-enriched irrigation water for the continuous irrigation of green beans, cabbage, potatoes and tomatoes on three different soil types in order to investigate its effects on biomass production and on the concentration of Se and other elements.

The aim of the experiment was to model the effects of elevated Se levels in the irrigation water and of soil texture on the yield and elemental composition of potato, cabbage, tomato and green bean plants. The recent work of Newman et al. [42] also drew attention to the human health implications of examining such data. In addition to the elemental composition, the fitness of the plants was examined by measuring the photosynthetic parameters: chlorophyll content index (CCI) and chlorophyll fluorescence (Fv/Fm), which are good indicators of the physiological effect of Se treatment. Based on the above, the following hypotheses were investigated:

(H1) Dissolved Se applied with irrigation water can theoretically easily reach the roots and be absorbed by the plant. Nevertheless, the uptake of Se may be influenced by soil factors, so it is important to examine the extent of this effect. For this reason, three field soils differing significantly in pH, clay and organic matter (OM) content were used in the pot experiment so that the effect of different soil properties on the fate of selenium could also be investigated. Selenium was expected to be more mobile and available for the test plants on soil with lower clay and OM contents but with a higher pH. (H2) It was expected that the Se treatment up to 500 µg L^−1^ Se concentration in irrigation water would not have a significant negative effect on plant biomass production but that the Se content of the plant tissues would increase. (H3) Se can be enriched in edible plant parts without a significant negative effect on the concentration of other elements, and the vegetables grown can be used as functional foods.

## 2. Results

### 2.1. Soil Elemental Composition

The original plant-available Se contents of the sand, silty sand and silt soils were 0.009, 0.016 and 0.010 mg·kg^−1^, respectively (see Materials and Methods), which increased 4–10 times in the Se-1 treatment (0.053, 0.065 and 0.097 mg·kg^−1^) and 26–47 times in the Se-2 treatment (0.290; 0.419 and 0.472 mg·kg^−1^), respectively. The plant-available Se contents in all three soils only showed a significant difference in the Se-2 treatment compared to the control, but a considerable increase could already be observed in the Se-1 treatment.

### 2.2. Biomass Production

The average biomass production and dry matter content of the vegetable crops are shown in Table 1. The Se treatments did not cause any significant changes in the biomass of the plants. However, the fresh weight of green bean, cabbage head, potato tuber and tomato fruit decreased non-significantly by 3.5, 6.4, 6.0 and 19.9% in Se-1 and by 12.8, 11.8, 8.0 and 8.9% in Se-2, respectively, compared to the control averaged over the three soils. At the same time, the dry matter content increased significantly in cabbage heads (Se-0: 8.9 ± 0.9a, Se-1: 11.2 ± 1.7b, Se-2: 11.2 ± 1.5b) and tomato fruit (Se-0: 6.67 ± 0.65a, Se-1: 7.39 ± 0.85b, Se-2: 7.87 ± 0.69b) as the Se dose increased (Supplementary Tables S1–S4).

### 2.3. Maximum Quantum Efficiency of PSII (Fv/Fm) and Chlorophyll Content Index (CCI)

In the case of tomato, potato and green bean, neither the soil texture nor Se treatments caused a significant difference in Fv/Fm (Table 2). The Fv/Fm ratio showed the highest values in cabbage leaves, but these values decreased at the higher Se dose, especially in sand. Changes in CCI due to Se addition depended on both the soil texture and the test plant (Table 2). Compared to the control plants, tomatoes grown in silty sand or silt and potatoes grown in sand or silty sand soil responded with significantly increased CCI values. In the case of green bean, the CCI values decreased in the Se-1 treatment in sand, but neither Se-1 nor Se-2 resulted in any change in silty sand or silt. Se had no detectable effect on the chlorophyll content of cabbage.

### 2.4. Elemental Concentrations of the Edible Parts of Vegetables

#### 2.4.1. Se Content

The relatively low Se concentration of 0.024–0.083 mg kg^−1^ DW in green beans on control soils increased 72–202-fold in the Se-1 treatment and 667–1732-fold in the Se-2 treatment, depending on the soil type (Table 3). Although the changes were already evident as a result of the Se-1 treatment, a significant difference compared to the control was only detected when the three soils were averaged, whereas the difference caused by the Se-2 treatment was significant on all three soils. In the Se-2 treatment, the Se concentration was significantly higher for sand than for the other two soil types. The Se content was also the highest in sand in the Se-1 treatment, but the difference was not significant.

In cabbage leaf, the Se content increased by approximately two orders of magnitude in the Se-1 treatment and by three orders of magnitude in the Se-2 treatment. The Se concentration was the highest on sand, but the difference was only significant in the Se-2 treatment.

In potato tubers, the Se content increased 11–72-fold in the Se-1 treatment, and 61–396-fold in the Se-2 treatment, but a significant difference was only detected between the control and the Se-2 treatment. The highest values were obtained in sand, but the differences were not significant.

The Se-1 treatment resulted in a 24–50-fold difference and Se-2 in a 124–214-fold difference in the Se content of tomato fruit, both of which were significant, as was the difference between the Se-1 and Se-2 treatments. The Se content was significantly lower in sand in the Se-2 treatment compared to the other soil types.

#### 2.4.2. Contents of Other Elements

**Figure 1:**
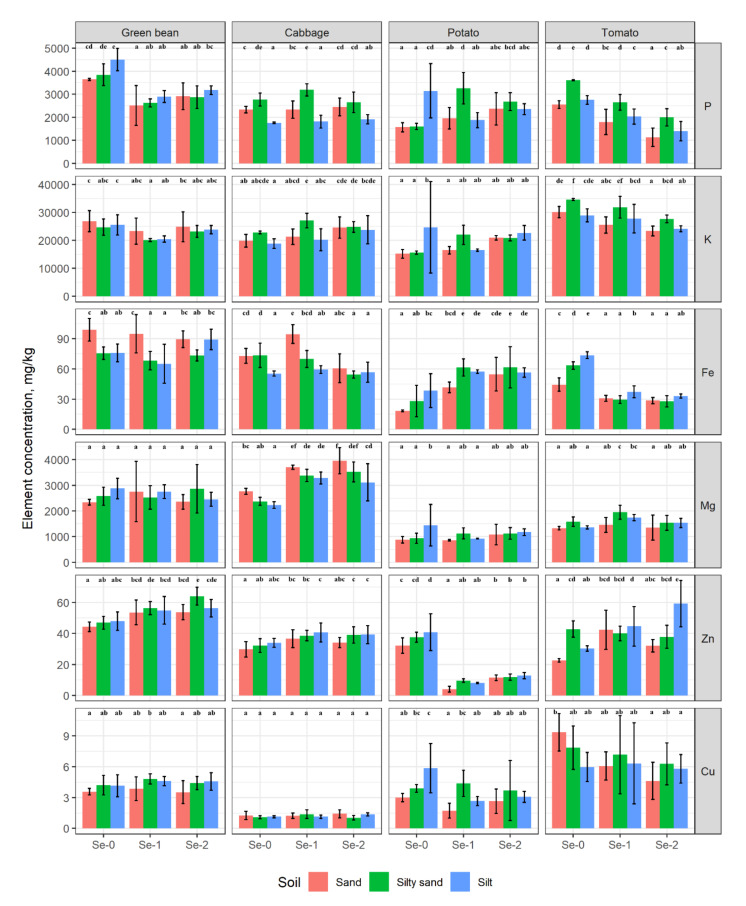
Selected macro- and micro elemental concentrations of the plants, mg·kg^−1^ DW. Different letters indicate significant differences between treatments (*p* < 0.05). Error bars represent standard deviations.

The P content in green beans consistently decreased as a result of Se treatment, but it was only significant compared to the control, while no significant difference could be detected between the Se-1 and Se-2 treatments. In cabbage leaf, the P content was primarily influenced by the soil type and exhibited little response to Se treatment. The P content of potato tubers increased significantly in silty sand but decreased in silt in the Se-1 treatment compared to the control, whereas in tomato fruit, it decreased significantly with increasing Se dose (Figure 1).

The K content showed a significant decrease in green beans in the Se-1 treatment in silty soil. The Se-2 doses increased the K content in cabbage leaf in sand and silt, whereas the control K values were the lowest. The changes in K content in potato were inconsistent, but it decreased significantly in tomato fruit in all the soils in response to either Se-1 or Se-2 treatment.

The Fe content showed no substantial change in green beans and was primarily influenced by the soil type, whereas in cabbage, it significantly decreased in silty sand in the Se-2 treatment and increased in sand in the Se-1 treatment compared to the control. In potato tubers, the Fe content tripled in sandy soil and more or less doubled in silty sand and silty soil as a result of the Se-2 treatment, but it decreased significantly in tomato fruit in all the soils compared to control.

The Mg content showed no substantial change in green beans, but it was significantly higher in cabbage leaf in all the soils as the Se dose increased and in tomato fruit in silty sand and silt soils in the Se-1 treatment. However, a decrease was observed in silt soil in the Se-1 treatment.

The Zn content showed an increasing trend in green beans, cabbage and tomato, but this change was only significant in green beans and cabbage in the lighter textured soils, while in tomato, it was significant in sand and silt soils. In potato tubers, the Zn content was significantly reduced compared to the control in all the soils.

The Cu content was unaffected by Se treatment in either the green beans or the cabbage, but it was significantly reduced in potato compared to the control in all the soils. A decrease was also observed in tomato, which was significant in sandy soil (Figure 1).

In the plant element content studies, statistically verifiable or trend-like changes were found in the elements described above. The As, B, I, Mn and Ca contents were also analyzed, but they were independent of the treatments, so no detailed description of these has been given. On average the edible parts of green beans, cabbage, potatoes and tomatoes contained 0.010, 0.089, 0.015, 0.007 mg kg^−1^ As; 17.4, 21.2, 6.61, 12.8 mg kg^−1^ B; 0.07, 1.15, 0.829, 0.088 mg kg^−1^ I and 19.5, 28.1, 4.85, 8.72 mg kg^−1^ Mn, respectively, in DW.

### 2.5. Selenium Content of Edible Parts in Relation with the Recommended and Toxic Selenium Intake

The Se contents of 100 g of fresh edible parts of each vegetable are given in Table 4. Cabbage had by far the highest Se content, followed by green beans, potatoes and tomatoes. Averaged over the three soils, the Se-1 treatment caused a 98-fold increase in Se content in beans, a 118-fold increase in cabbage, a 19-fold increase in potatoes and a 39-fold increase in tomatoes compared with the untreated controls, while the Se-2 treatment induced 809, 735, 99 and 195-fold increases, respectively.

Consuming 100 g of beans given the Se-1 treatment would cover the daily need for Se, while the same amount of potatoes or tomatoes would provide approximately 70–80% of the recommended dietary allowance (RDA). In the case of cabbage, however, 100 g would be equivalent to four times the RDA value. The Se-2 treatment resulted in Se contents higher than the RDA in all cases, with a 26-fold value for cabbage (Table 5).

Table 6 compares the daily intake with the tolerable upper intake level, which is also known as the hazard quotient (HQ), expressed as a percentage. The amount of Se ingested with 100 g of fresh vegetables in Se-1-treated foods remained below the tolerable upper intake level (UL) without exception; cabbage approached this level to the greatest extent, with a value of just over 50%. In the Se-2 treatment, on the other hand, green beans reached the UL value and cabbage exceeded it more than 3 times.

## 3. Discussion

### 3.1. Biomass Production

Both soil type and Se treatment influenced the biomass production of certain parts of the vegetables in some cases, but no interaction was detected between soil type and Se treatment. In terms of the edible plant parts, i.e., green beans, tomato fruit, potato tubers and cabbage heads, there was a slight decrease in fresh yield as a result of Se treatment, but this effect was not significant.

Although the decrease in green bean biomass was not significant after Se treatment, it was in agreement with the findings of Figueiredo et al. [43], who treated three different varieties of beans (*Phaseolus vulgaris*) with 5 μM Na_2_SeO_4_ in hydroponic culture and observed a decrease in the fresh and dry mature seed weight of two varieties, and an increase in one.

Se treatment may have a growth-promoting effect on cabbage in a hydroponic culture [44]. Zhao et al. [45] treated black cabbage (*Brassica campestris*) with 2 mg kg^−1^ Se in a pot experiment involving different Se^6+^-Se/Se^4+^-Se ratios. They found that the yield was reduced as the Se^6+^-Se content rose to over 0.5 mg kg^−1^ soil and fell to a quarter at 2.0 mg kg^−1^. In the present experiment, the Se content of the soil remained below 0.5 mg kg^−1^, and no biomass decrease was observed.

Regarding the effect of Se treatment on the potato yield, the results were similar to those given in the literature. Lei et al. [46] found a negative correlation between increasing Se concentrations in the soil and the fresh weight of red potato tuber. However, as Se stimulated tuber formation, there was a larger number of smaller potatoes. Kádár and Prokisch [47] applied extremely high doses of 90, 270 and 810 kg ha^−1^ Se in the form of Na_2_SeO_3_ in a field experiment, resulting in 7, 66 and 81 mg kg^−1^ (resp.) ammonium-acetate-lactate soluble Se concentrations in the soil, accompanied by a 16, 71 and 88% loss, respectively, in fresh potato tuber yield. Oliveira et al. [48] also found that above 0.75 mg/kg Se content in soil, the tuber production of potato showed a significant decrease.

The yield data for tomato fruit showed only minor changes, so the 0.1 and 0.5 mg Se L^−1^ doses caused no significant effects; this was also true for root and stem biomass. This was confirmed by Edelstein et al. [24], who found no significant decrease in tomato yield up to a dose of 0.5 mg Se L^−1^ (as Na_2_SeO_4_) in the irrigation water in plants grown on perlite. At 1.5 mg Se L^−1^, the yield was only 51% of the control. However, when Foroughbakhch Pournavab et al. [25] used Na_2_SeO_3_ to enrich the irrigation water with 2 or 5 mg Se L^−1^, the yield parameters improved for both the stem and fruit of tomato grown in peat-moss or perlite substrate, though the differences were only significant for the stem.

### 3.2. Changes in the Chlorophyll Fluorescence Parameter Fv/Fm and the Chlorophyll Content Index (CCI)

One of the aims of the research was to evaluate the physiological responses of green bean, tomato, cabbage and potato to Se treatments, thus supporting development of the biofortification by optimal Se level in irrigation water. Chlorophyll fluorescence and leaf chlorophyll content are the most widespread, physiology-based indicators of environmental stresses [49]. A low concentration of supplementary selenium in the growth medium may improve the physiological processes of plants [12,15], especially under abiotic stress conditions. The presence of 1μM selenite or selenate ameliorated the photosynthesis of tomato exposed to Cd contamination [16]. Moreover, 10 μM of selenite increased the photosynthesis, stomatal conductance and transpiration of tomato [50]. Diao et al. [51] demonstrated the improved photochemical efficiency of PSII in Se-treated (Na_2_SeO_3_ 0.05 mM) tomato. Numerous studies have shown that the application of Se in low concentrations may increase the chlorophyll (a + b) and carotene content in cabbage leaves [12], maize [52] and faba bean [53]. The present experiment did not confirm the beneficial effect of Se treatment on chlorophyll fluorescence in general, as even 500 µg Se L^−1^ in the form of Na_2_SeO_4_ decreased the Fv/Fm of cabbage compared to both the control and the 100 µg Se L^−1^ dose (Table 2). The leaf photochemical efficiency (Fv/Fm) changed within a small range from 0.714 to 0.833, and its high values indicated a good physiological condition of the test plants. The leaf greenness (measured as CCI value) was a sensitive indicator, not only to Se treatments, but to soil textures and to plant species as well. The highest CCI values in silt soils could be caused by its higher nitrogen supply. As reported by other authors [12], Se addition may significantly increase the CCI chlorophyll level in leaves, except for bean and cabbage.

### 3.3. Changes in the Se Concentrations of the Vegetables

When interpreting changes in elemental contents, it is important to note that they are often influenced by biomass production. As noted above, in most cases Se slightly reduced the biomass, which may have contributed to the higher concentrations of elements, known as the concentration effect [54].

For vegetables, the primary consideration is the Se content of the edible parts. In the present experiment, the 0.02–0.08 mg kg^−1^ Se concentration in untreated beans (see Table 3) was broadly consistent with data from other experiments. The Se content of different varieties of bean seeds increased from around 0.1 μg g^−1^ to 2–4 μg g^−1^ as a result of 5 μM Na_2_SeO_4_ treatment [42]. Smrkolj et al. [55] sprayed the leaves of beans with Na_2_SeO_4_ at a concentration of 10 mg Se L^−1^ on two occasions, which increased the Se content from 0.05 µg g^−1^ (DW) of the control to 2 µg g^−1^. The applied concentration of 100 µg Se L^−1^ in irrigation water therefore has a stronger effect. However, Azhar-u-ddin et al. [56] set the Se concentration of soils to 1 and 4 mg Se kg^−1^, applied in the form of selenate, which resulted in a bean fruit Se concentration of approximately 10 and 20 mg kg^−1^, respectively, which exceeds the values obtained in Se-1 treatment but does not reach that of Se -2 treatment.

Cabbage belongs to the *Brassicaceae* family, which is characterized by the ability to accumulate Se in an organic form [57]. Being a secondary accumulator, cabbage may have a Se contents as high as 100–1000 mg kg^−1^ DW, whereas bean, potato and tomato are non-accumulators [15]. Funes-Collado et al. [58] also found that among the vegetables studied (cabbage, lettuce, chard and parsley), cabbage had the highest Se content. This is consistent with the trends observed in this experiment, as the concentration of Se in the cabbage head is several times higher than in the other three vegetable plants.

With Se-1 and Se-2 treatments, 1.07 and 5.35 mg Se per pot were applied, respectively, which increased the Se content of potato from 0.11 to 2.2 and 11.4 mg kg^−1^, respectively. A larger increase was reported by Turakainen et al. [59], who applied a total of 0.75 mg and 3 mg Se in the form of Na_2_SeO_4_ to pots containing 10 kg of pure quartz sand, which increased the Se in potato stolon from less than 1 mg kg^−1^ to about 13 and 40 mg kg^−1^ (resp.). Kádár and Prokisch [47] examined the effect of 90, 270 and 810 kg ha^−1^ Se treatment, which increased the 3.4 mg kg^−1^ Se content of the control to 46.9, 84.0 and 75.4 mg kg^−1^, respectively, in potato tubers. According to Öborn et al. [60], the Se content of potato positively correlates with soil pH, the tendency of which can be observable in our experiment (Table 3), although the differences were not significant due to the close pH values of the investigated soils.

In the present experiment, the 0, 0.1 and 0.5 mg L^−1^ Se applied in the form of Na_2_SeO_4_ resulted in Se contents of 0.14, 4.99 and 23.3 μg g^−1^ (DW) in tomato fruit (Table 3). However, in the case of the Se content measured at the time of harvest in tomatoes, it must be considered that the Se content may decrease during post-harvest ripening [61]. Pezzarossa et al. [62] found a very similar result when treating hydroponically grown tomatoes with 1 mg Se L^−1^ in the form of Na_2_SeO_4_ in the nutrient solution, which increased the fruit Se concentration from 0.1 (control) to 10 μg Se g^−1^ (DW), equivalent to 0.58 µg g^−1^ Se content in the fresh fruit. Edelstein et al. [24], on the other hand, measured an already high content of 80 μg Se g^−1^ in control tomato fruit, which increased approximately three times, i.e., to 230 μg Se g^−1^ (DW), when irrigation water contained 1.5 mg L^−1^ selenium as Na_2_SeO_4_.

In summary, the Se content of treated potato tubers was relatively low compared to the other plants, whereas cabbage leaves were more efficient in absorbing selenium from the soil.

The Se content of edible plant parts was generally the highest in sandy soil. This may be because the clay content of the soil binds selenium, making it less available. Ajwa et al. [63] also found that plants absorbed less Se in soils with higher clay content compared to looser soils. Zhao et al. [45], however, found that the available Se content was more closely correlated with the silt content than the clay content, although his results showed that the dominant factor was the CaCO_3_ content, with a correlation coefficient above 0.8. In the present experiment, the coarse-textured sandy soil had the highest CaCO_3_ content, so this may have facilitated the increased Se uptake on this soil.

### 3.4. How Does Se Affect Other Elements in Vegetables?

In the present study, Se treatment was found to have a significant effect on the element composition of the edible parts of the vegetables investigated (Figure 1).

P content underwent frequent and inconsistent changes. Selenite may suppress P uptake and transport in plants, as found in the case of rice and Chinese brake fern [41,64], but it may also have no effect [25]. One reason for Se and P antagonism may be the competition between the SeO_3_^3−^ and PO_4_^3−^ anions [65]. The interaction depends also on the concentration. The addition of P to growth media with low Se concentrations increased the plant Se content up to a certain concentration and then decreased it, while in the case of a medium with higher Se content, the plant Se content continuously increased with increasing P doses [66]. However, in this present experiment, Se was applied in the form of selenate, which was not reported to affect P uptake, and the conversion of selenate to selenite is also unlikely under the experimental conditions used.

Data in the literature on the relationship between Se treatment and plant K content are contradictory, as also reflected in the present results. When Drahoňovský et al. [67] investigated the effect of foliar Se application on 12 plant species, the changes observed in K contents were diverse. K plays a significant role in the regulation of osmotic pressure in the cells. Pazurkiewicz-Kocot et al. [68] assumed that changes in the K content of maize (*Zea mays*) organs after Se application could be attributed to alterations in cell membrane permeability caused by Se. In contrast to the present results, Zhu et al. [69] observed a slight increase in the K content of tomato after leaf spraying with 1 mg Na-selenate per plant. Similarly, Foroughbakhch Pournavab et al. [25] found that adding Se to the irrigation water in the form of Na-selenite increased the K content in tomato fruit, but Edelstein et al. [24] reported that Se had no effect of on the K content of tomato fruit when 1.5 mg/L Na-selenate solution was used for irrigation. Similarly, Narváez-Ortiz et al. [70] found no K content changes in tomato fruit after applying Se in fertilizer solution or foliar spraying. In these studies, the tomato fruit biomass was not or was negatively affected by Se treatment, while in the present experiment, Se treatments resulted in an increment in fruit biomass, which may have caused the lower K content (dilution effect). However, in the case of cabbage, the K content in the leaves increased in parallel with the increase in biomass. No direct data were found in the literature on the effect of Se on the K content of cabbage, but the present results contradicted with the findings of Ulhassan et al. [71], where Se treatment had a negative effect, if any, on K uptake and translocation in various *Brassica napus* cultivars. In potato tubers a combination of Se and iodine was reported to increase the K content [72], but in the present experiment, treatment with Se was not effective in this respect.

In this study, Fe showed a significant increase in potato, but there was no clear tendency in the other plants. The Fe content in the human diet is a global issue. Iron deficiency, the most frequent cause of anemia, affects the population worldwide [73], so the significant increment observed in the Fe content of potato is favorable. To the best of our knowledge, the effect of Se on the potato Fe content has not yet been studied. Although tomatoes belong to the same genus, the direction of Fe content change was the opposite to that in potato. As in the present experiment, Zhu et al. [69] reported a non-significant reduction in the Fe content of tomato fruit after leaf spraying with Na-selenate. However, Schiavon et al. [74] only found a reduced Fe content in the root tissue of hydroponically grown tomato treated with 5 μM selenium. The decrease in cabbage Fe content in silty sand was in agreement with the findings of He et al. [75], where 1 mg/kg Se addition to the soil halved the Fe content of Chinese cabbage (*Brassica rapa*). These diverse results indicate the need for a deeper exploration of the effect of Se on Fe uptake in light of reports on the key role of Se in plant Fe uptake [15].

Se treatment only had a significant influence on the Mg content in cabbage, where an increment was observed. In contrast, He et al. [75] found that the Mg content of Chinese cabbage and lettuce (*Lactuca sativa*) remained unchanged after Se treatment. The inconsistent changes in the Mg content of tomato fruit in the present experiment are reflected in the literature, where Castillo-Godina et al. [76] detected an increase, Edelstein et al. [24] detected a decrease and Foroughbakhch Pournavab et al. [25] detected no change in the Mg content of tomato after Se treatment. Regarding potato, Wang et al. [77] found no changes in Mg content after Se fertilization in soil, similar to our results observed in lighter textured soils. According to Newman et al. [42], the diversity of the results could also be traced back to differences in the experimental methods applied.

The biofortification of food plants with Zn is already an important food industry goal in itself [78], so it is gratifying that Se tended to improve the Zn content of edible plant parts, with the exception of potatoes. Since an increase in P and a concomitant decrease in Zn content were observed in potato, compared with lower P and higher Zn content in green bean, one possible reason for this phenomenon could be antagonism between P and Zn [79]. Boldrin et al. [64] found that Se treatment decreased the P content but significantly increased the Zn content in rice. However, in cabbage, the Zn content increased in parallel with P. He et al. [75] detected a very slight, non-significant decrease in the Zn content of Chinese cabbage and lettuce plants treated with 1 mg kg^−1^ Se, but P was not measured. However, Dai et al. [80] found that spiking soil with Se enhances Zn accumulation in Chinese cabbage plants.

In some cases, the Cu content decreased in potato and tomato, which is consistent with the results of other research. Schiavon et al. [74] also found reduced Cu content in tomato roots as a result of 10 μM Se treatment. He et al. [75] found a significant decrease in the Cu content after Se treatment in Chinese cabbage and lettuce. Similarly, in a study of 12 plant species, Drahoňovský et al. [67] observed that Se treatment significantly modified the Cu content of the plants in only five species and caused a decrease in four of them.

### 3.5. Biofortification and Consumability of Functional Foods

The recommended dietary allowance (RDA) of Se ranges from 25 to 60 µg day^−1^ for adult women and from 30 to 75 µg day^−1^ for adult men, depending on countries or regions. The gap between deficient and toxic amounts is relatively small. The tolerable upper intake level of Se is only 350–450 µg day^−1^ [4]. The estimated daily intake in Eastern European countries is 30–40 µg Se day^−1^, which is slightly below the recommended intake level [81].

Based on the present results, the consumption of 100 g of fresh edible plant parts of potato and tomato provides close to the 55 µg RDA for Se in the EU, USA and Canada, while in the case of green bean, the RDA level is reached [4]. Thus, fertigation with 100 µg Se L^−1^, the concentration used in the Se-1 treatment, could be a satisfactory solution for these vegetables, and functional foods grown with this method would not be harmful to health but would compensate for low Se intake (Table 5). However, even this level of biofortification results in Se values 4–4.5 times greater than RDA in cabbage heads, which is also half of the tolerable upper intake level, so in this case, the concentration of Se in the irrigation water should definitely be below 100 µg L^−1^ (Table 6). It is important to know that heat treatment may cause a loss of Se from food or transform Se speciation [82]. In the case of garlic, the more bioavailable organic Se fractions decreased due to protein denaturalization as a result of boiling, baking, microwaving and frying [83]. In contrast, boiling increased the SeMet concentration and reduced the Se(VI) content of cabbage [84]. The present results confirmed that the 20 µg Se L^−1^ upper limit commonly used for irrigation water is safe.

The Se-2 treatment resulted in an undesirable increase in the concentration, so the use of water with a 500 µg Se L^−1^ concentration for irrigation is not recommended for biofortification. It is necessary to note that in addition to the concentration of Se in the irrigation water, the amount of water irrigated, based on the water demand of the plant, determines the amount of total Se applied during the growing season of the plants, which also contributes to the results. Materials and Methods provide information on the total amount of Se applied to each plant in each treatment.

## 4. Materials and Methods

### 4.1. Experimental Materials and Conditions

The effect of Se-enriched irrigation was studied on green bean (*Phaseolus vulgaris* L., cv. Golden Goal), cabbage (*Brassica oleracea* L. var. *capitata* cv. Zora), tomato (*Solanum lycopersicum* L. cv. Mano) and potato (*Solanum tuberosum* L. cv. Balatoni rózsa) in an open greenhouse at the Experimental Station of the Center for Agricultural Research in Őrbottyán, Hungary [85,86,87]. A pot experiment was performed using the top 0–20 cm layer of soils from three different locations in Hungary, all having distinct properties: sand (Mollic Umbrisol (Arenic) from Őrbottyán), silty sand (Luvic Calcic Phaeozem from Gödöllő) and silt (Calcic Chernozem from Hatvan) [88]. The basic characteristics of the air-dry soils are shown in Table 7, and total and mobilizable element concentrations of the soils are shown in Supplementary Table S5.

Four holes, each 0.5 cm in diameter, were drilled in the bottom of the 10 L pots to allow leached water to escape. The bottom of each pot was filled with gravel with a diameter of 4–8 mm in a 1 cm layer, which was covered with a fine synthetic fiber fabric and 10 kg of soil.

The cabbage and tomato seeds and the potato tubers were first germinated, then planted and grown for three weeks in a commercial growth medium (Vegasca Bio soil mix; Florasca Hungary Ltd.; mixture of peat and grey cattle manure compost. OM > 50%; N > 0.3%; P_2_O_5_ > 0.1%; K_2_O > 0.1%; pH of 6.8) under controlled climatic conditions in a growth chamber (16/8 h photoperiod, 25–27/15–17 °C temperature and 600 μmol/m^2^/s photon flux density). Soil-free seedlings were transplanted into plastic pots (1 seedling/pot) after a 6-day acclimatization period in the greenhouse. Germinated seeds of green bean were planted directly into the soils (1 seed/pot).

The Se-enrichment of the irrigation water started three weeks after planting. The experiment was set up in three replicates on three soil types and included three treatment levels: Se-0: control, Se-1: 100 and Se-2: 500 µg Se L^−1^ in the form of Na_2_SeO_4_ in the irrigation water. These concentrations are an order of magnitude higher than the recommended values for irrigation water by FAO [89]. The water was stored in 0.5 m^3^ tanks before the application. Irrigation was carried out with an automatic irrigation system, with individual drip stakes placed in each pot. The daily volume of irrigation water was adjusted according to the requirements of the plants: soil moisture content was monitored at a depth of 10 cm every hour (Sensor: Decagon EC-5). The nutrient requirements of all the treated plants were met during the vegetation period using 200 mL Hoagland solution per pot, applied weekly by hand. The total number of plants included in the experiment was 3 Se levels × 3 soil types × 4 plant species × 3 replicates = 108 plants. Details of the growth period and irrigation are shown in Table 8.

The plants received natural sunlight in the greenhouse. Climate data were monitored throughout the growth period, as reported in Table 9. Pesticide (Decis, Bayer) was applied whenever necessary.

### 4.2. Sample Preparation, Analysis and Measurements

The plants were washed with deionized water after harvest; then, the plant parts were separated (root, tuber, leafy shoot, fruit), and the fresh biomass was weighed. Subsequently, the roots and aerial parts of the plants were dried at 40 °C in a laboratory dryer for two days, except for tomato and green bean fruits and potato tubers, which were milled and freeze-dried at −70 °C, 200 Pa for 72 h, before measuring the dry mass of the plant organs. Dried samples were homogenized with a blending machine, equipped with plastic housing and stainless-steel blade. The dried, homogenized samples were mineralized in a microwave-assisted acid digestion system (TopWave, Analytik Jena, Germany). Plant samples of 400–500 mg were digested in a mixture of 7 mL 67% HNO_3_ (VWR International, Radnor, PA, USA) and 3 mL 30% H_2_O_2_ (VWR International, PA, USA). After digestion, internal standards (Sc, Y, In) were added to the solutions, which were made up to 15 mL with deionized water.

The concentrations of selenium, macro- and micro-elements in the dry weight (DW) of plant samples were measured using an inductively coupled plasma mass spectrometer (ICP-MS) (PlasmaQuant Elite, Analytik Jena, Germany).

Composite soil samples were collected from the soils to analyze their basic parameters, and soil samples from each pot were also analyzed after removing the plant residues. The soil samples were dried and sieved through a 2 mm mesh sieve. The pH was measured according to the Hungarian Standard Method [90] in a 1:2.5 soil:water suspension 12 h after mixing, and the OM content was measured using the modified Walkley–Black method [91]. The total N content was measured with the Kjeldahl method [92], and the NH_4_-N and NO_3_-N concentrations from KCl extracts were measured according to the Hungarian Standard [93]. The CaCO_3_ content was measured using the Scheibler gas-volumetric method [90], and the CEC values were measured with the modified method of Mehlich [94]. Plant-available P and K concentrations were determined after extraction with ammonium acetate-lactate (AL-P_2_O_5_ and AL-K_2_O) [95]. Plant-available Se and other element contents were measured in 0.5 M NH_4_-acetate + 0.02 M EDTA extract according to Lakanen and Erviö [96] (referred to as LE). The total Se concentrations were determined from the samples using aqua regia in a microwave teflon bomb [97]. The element contents of the soil samples were analyzed using the ICP-MS instrument.

To detect plant physiological responses to Se treatments, the photochemical activity of the photosystem (PS) II (Fv/Fm) of dark adapted leaves (15 min) was measured as the chlorophyll *a* fluorescence intensity (Opti-Sciences OS-30p+ Fluorometer, Hudson, New Hampshire, USA). F_v_/F_m_ values were calculated by the method of Tsimilli–Michael and Strasser [98]. The chlorophyll content index (CCI) of the leaves was detected with a Chlorophyll Content Meter (CCM-200 plus, Opti-Sciences, Hudson, NH, USA). The F_v/_F_m_ and CCI values were measured on the youngest adult leaves at the harvesting stage for all the plants.

### 4.3. Statistical Analysis

A two-factor factorial analysis of variance was used to evaluate the plant parameters, one factor being the soil type and the other the Se dose. The level of significance was set to a 95% confidence interval (*p* < 0.05). Significantly different groups were determined using Tukey’s HSD post hoc test. Statistica v.13 (StatSoft Inc.) software was used for all the statistical calculations. Data visualization was made with *R* statistical software [99] using a *ggplot2* package [100].

## 5. Conclusions

Irrigation water enriched with Se significantly increased the plant-available Se content of the soil, which was lower in sandy soil and higher in silty sand and silt soils. Regarding the Se treatment’s effect on plant parameters, our main findings are the following:

The treatments caused a slight decrease in the biomass production of the edible parts of green bean, cabbage, potato and tomato, while the dry matter content of cabbage heads and tomato fruit increased.

Se had a beneficial effect on potato and tomato CCI values, with a significant increase in this parameter in plants.

Se treatment had the greatest effect on the Se content of the edible parts of the plants, which increased by several orders of magnitude, while P, K, Fe, Mg, Zn and Cu increased or decreased depending on the soil types or plant species. Se treatments decreased the P and K contents in green beans, the Zn and Cu contents in potato and the P, K, Fe and Cu contents in tomato. Se treatments had a positive effect on Zn content in green beans; on K, Mg and Zn contents in cabbage; on Fe content in potato and on Zn content in tomato. In the other cases, the changes were different as a function of soil type, or the Se treatment had no effect.

With respect to the soil types, the Se content of the edible plant parts was the highest in sandy soil for most species and treatments.

Based on the Se content of the fresh edible plant parts and the recommended dietary allowance, 100 µg Se L^−1^ fertigation can be recommended for the production of functional foods for green beans, potatoes and tomatoes, but as cabbage accumulates more Se, irrigation water with a lower Se content is recommended. In the case of cabbage, a more accurate determination of the Se content, and in the case of foods typically consumed after processing, a more detailed examination of the effect of frying and cooking on the Se content could serve as topics for further research. The use of Se-enriched irrigation water might be a suitable method for Se biofortification without a significant reduction in plant biomass production and without a remarkable modification of other macro- and microelement contents.

## Figures and Tables

**Table 1:** Average biomass production (g·plant^−1^) and dry matter content (%) of the vegetable crops.

Parameter	Green Bean	Cabbage	Potato	Tomato
Root dry weight (g)	2.41 ± 0.90	2.40 ± 0.91	2.99 ± 0.78	3.07 ± 0.76
Shoot dry weight (g)	12.8 ± 2.7	-	9.51 ± 1.43	28.8 ± 5.7
* Edible part fresh weight (g)	99.7 ± 21.5	484 ± 102	183 ± 13	295 ± 87
Edible part dry weight (g)	10.5 ± 2.9	50.4 ± 12.2	36.0 ± 3.3	21.3 ± 5.8
Edible part dry matter content (%)	10.4 ± 1.1	10.4 ± 1.7	19.7 ± 1.6	7.31 ± 0.87

* Edible parts: green beans, cabbage heads, potato tubers, tomato fruit.

**Table 2:** Photosynthetic parameters: Effects of Se treatment on the chlorophyll fluorescence (Fv/Fm) and chlorophyll content index (CCI) of the test plants.

Plant	Parameter	Se		Soil Type		Mean
		Dose	Sand	Silty Sand	Silt	
Green bean	Fv/Fm	Se-0	0.735 ± 0.012 aA	0.764 ± 0.033 aA	0.773 ± 0.023 aA	0.758 ± 0.027 A
		Se-1	0.732 ± 0.018 aA	0.794 ± 0.024 aA	0.766 ± 0.049 aA	0.764 ± 0.039 A
		Se-2	0.741 ± 0.048 aA	0.768 ± 0.009 aA	0.722 ± 0.035 aA	0.761 ± 0.033 A
	CCI	Se-0	14.2 ± 2.9 aB	20.6 ± 1.5 aA	19.1 ± 4.1 aA	18.0 ± 3.9 A
		Se-1	12.3 ± 0.9 aA	20.4 ± 1.7 bA	21.3 ± 4.1 bA	18.0 ± 4.9 A
		Se-2	13.4 ± 0.9 aB	21.2 ± 1.5 bA	18.1 ± 2.9 bA	17.6 ± 3.8 A
Cabbage	Fv/Fm	Se-0	0.831 ± 0.003 aB	0.826 ± 0.012 aA	0.836 ± 0.002 aA	0.831 ± 0.008 B
		Se-1	0.829 ± 0.005 aB	0.833 ± 0.001 aA	0.837 ± 0.006 aA	0.833 ± 0.005 B
		Se-2	0.805 ± 0.019 aA	0.818 ± 0.005 aA	0.828 ± 0.005 aA	0.817 ± 0.014 A
	CCI	Se-0	20.9 ± 4.3 aA	24.4 ± 7.2 aA	33.0 ± 16.4 aA	26.1 ± 10.7 A
		Se-1	25.5 ± 0.9 aA	35.5 ± 9.7 aA	33.7 ± 5.1 aA	31.6 ± 7.2 A
		Se-2	28.5 ± 10.3 aA	28.5 ± 2.9 aA	33.3 ± 5.2 aA	30.1 ± 6.4 A
Potato	Fv/Fm	Se-0	0.667 ± 0.028 aA	0.734 ± 0.045 aA	0.726 ± 0.046 aA	0.709 ± 0.047 A
		Se-1	0.709 ± 0.044 aA	0.724 ± 0.065 aA	0.726 ± 0.039 aA	0.720 ± 0.045 A
		Se-2	0.706 ± 0.039 aA	0.717 ± 0.004 aA	0.720 ± 0.052 aA	0.714 ± 0.033 A
	CCI	Se-0	11.1 ± 1.0 aA	13.1 ± 2.2 aA	19.0 ± 2.0 aA	14.4 ± 3.9 A
		Se-1	13.7 ± 0.7 bB	10.7 ± 0.6 aA	22.9 ± 0.9 bA	15.8 ± 5.5 AB
		Se-2	15.4 ± 2.7 aB	13.6 ± 1.3 aB	24.1 ± 3.3 aA	17.7 ± 5.3 B
Tomato	Fv/Fm	Se-0	0.786 ± 0.009 aA	0.792 ± 0.006 aA	0.771 ± 0.025 aA	0.783 ± 0.017 A
		Se-1	0.784 ± 0.008 aA	0.778 ± 0.016 aA	0.797 ± 0.015 aA	0.786 ± 0.014 A
		Se-2	0.778 ± 0.017 aA	0.796 ± 0.011 aA	0.796 ± 0.006 aA	0.789 ± 0.015 A
	CCI	Se-0	12.5 ± 3.1 aA	10.9 ± 1.0 aA	16.0 ± 8.5 aA	13.1 ± 5.1 A
		Se-1	17.5 ± 4.2 aA	19.4 ± 1.7 abB	30.6 ± 8.2 bB	22.4 ± 7.7 B
		Se-2	18.5 ± 1.5 aA	29.8 ± 1.9 abB	34.3 ± 2.9 bB	27.5 ± 7.5 B

Means ± std. dev., lower case indicates significant differences between columns (soil types) and capitals between rows (Se doses) (Tukey HSD_5%_).

**Table 3:** Se concentrations of the edible parts * of vegetables as a function of Se treatments and soil types, mg·kg^−1^ DW.

Plant	Se		Soil Type		Mean
	Dose	Sand	Silty Sand	Silt	
Green bean	Se-0	0.083 ± 0.01 aA	0.024 ± 0.007 aA	0.067 ± 0.023 aA	0.058 ± 0.029 A
	Se-1	5.95 ± 0.78 aA	4.85 ± 0.31 aA	5.48 ± 0.69 aA	5.43 ± 0.72 B
	Se-2	55.4 ± 7.9 bB	41.7 ± 2.9 aB	41.8 ± 3.0 aB	46.3 ± 8.2 C
Cabbage	Se-0	0.189 ± 0.015 aA	0.167 ± 0.014 aA	0.283 ± 0.012 aA	0.213 ± 0.055 A
	Se-1	23.2 ± 0.5 aB	22.4 ± 1.1 aB	16.7 ± 1.1 aB	20.8 ± 3.2 B
	Se-2	150 ± 6 bC	116 ± 5 aC	119 ± 9 aC	128 ± 17 C
Potato	Se-0	0.205 ± 0.012 aA	0.111 ± 0.018 aA	0.027 ± 0.003 aA	0.114 ± 0.078 A
	Se-1	2.31 ± 0.22 aA	2.27 ± 0.05 aA	1.95 ± 0.03 aA	2.17 ± 0.21 B
	Se-2	12.6 ± 2.4 aB	11.0 ± 1.4 aB	10.7 ± 2.3 aB	11.4 ± 2.0 C
Tomato	Se-0	0.101 ± 0.008 aA	0.126 ± 0.015 aA	0.194 ± 0.001 aA	0.140 ± 0.042 A
	Se-1	5.06 ± 0.14 aB	5.05 ± 0.25 aB	4.85 ± 0.18 aB	4.99 ± 0.20 B
	Se-2	21.6 ± 1.4 aC	23.5 ± 1.0 bC	24.8 ± 0.5 bC	23.3 ± 1.7 C

Means ± std. dev., lower case indicates significant differences between columns (soil types) and capitals between rows (Se doses) (Tukey HSD_5%_); * green beans, potato tubers, cabbage leaves, tomato fruit.

**Table 4:** Se content of edible parts * of vegetables, µg 100 g^−1^ in fresh weight.

Plant	Se		Soil Type	
	Dose	Sand	Silty Sand	Silt
Green bean	Se-0	0.767 ± 0.026 aA	0.263 ± 0.056 aA	0.715 ± 0.270 aA
	Se-1	55.4 ± 10.7 aA	55.9 ± 3.6 aA	59.7 ± 6.5 aA
	Se-2	493 ± 64 aB	454 ± 51 aB	465 ± 50 aB
Cabbage	Se-0	1.60 ± 1.18 aA	1.37 ± 0.15 aA	2.85 ± 0.23 aA
	Se-1	252 ± 22 aA	222 ± 41.5 aA	215 ± 33 aA
	Se-2	1551 ± 261 aB	1347 ± 215 aB	1383 ± 14 aB
Potato	Se-0	4.07 ± 0.31 aA	2.12 ± 0.286 aA	0.530 ± 0.030 aA
	Se-1	46.4 ± 4.6 aB	43.1 ± 2.9 aB	39.4 ± 0.7 aB
	Se-2	230 ± 19 aC	226 ± 16 aC	209 ± 15 aC
Tomato	Se-0	0.680 ± 0.086 aA	0.791 ± 0.063 aA	1.36 ± 0.20 aA
	Se-1	32.3 ± 1.5 aB	40.6 ± 2.3 aB	37.5 ± 2.3 aB
	Se-2	157 ± 10 aC	186 ± 25 bC	210 ± 13 bC

Means ± std. dev., lower case indicates significant differences between columns (soil types) and capitals between rows (Se doses) (Tukey HSD5%); * see Table 3.

**Table 5:** Amount of Se ingested with 100 g of fresh edible vegetable parts * as a percentage of the recommended dietary allowance (RDA) **.

Plant	Se		Soil Type	
	Dose	Sand	Silty Sand	Silt
Green bean	Se-0	1.40 ± 0.05	0.479 ± 0.102	1.30 ± 0.49
	Se-1	101 ± 20	102 ± 7	109 ± 12
	Se-2	897 ± 116	826 ± 94	845 ± 91
Cabbage	Se-0	2.92 ± 0.32	2.49 ± 0.27	5.19 ± 0.42
	Se-1	458 ± 41	403 ± 75	391 ± 60
	Se-2	2820 ± 475	2449 ± 391	2514 ± 25
Potato	Se-0	7.40 ± 0.56	3.85 ± 0.52	0.963 ± 0.055
	Se-1	84.3 ± 8.5	78.3 ± 5.3	71.7 ± 1.3
	Se-2	419 ± 35	410 ± 29	381 ± 27
Tomato	Se-0	1.24 ± 0.16	1.44 ± 0.12	2.47 ± 0.37
	Se-1	58.8 ± 2.8	73.9 ± 4.1	68.1 ± 4.1
	Se-2	285 ± 18	338 ± 45	381 ± 24

* see Table 3; ** Based on 55 µg Se RDA in the EU, USA and Canada (Fairweather-Tait et al., 2011).

**Table 6:** Amount of Se ingested per 100 g of fresh edible vegetable parts * as a percentage of the tolerable upper intake level (UL) **.

Plant	Se		Soil Type	
	Dose	Sand	Silty Sand	Silt
Green bean	Se-0	0.171 ± 0.006	0.059 ± 0.012	0.159 ± 0.060
	Se-1	12.3 ± 2.4	12.4 ± 0.8	13.3 ± 1.4
	Se-2	110 ± 14	101 ± 11.4	103 ± 11
Cabbage	Se-0	0.356 ± 0.039	0.304 ± 0.033	0.634 ± 0.051
	Se-1	55.9 ± 5.0	49.2 ± 9.2	47.8 ± 7.4
	Se-2	345 ± 58	299 ± 48	307 ± 3
Potato	Se-0	0.904 ± 0.069	0.471 ± 0.063	0.118 ± 0.007
	Se-1	10.3 ± 1.0	9.58 ± 0.65	8.76 ± 0.16
	Se-2	51.2 ± 4.2	50.1 ± 3.5	46.5 ± 3.3
Tomato	Se-0	0.151 ± 0.019	0.176 ± 0.014	0.302 ± 0.045
	Se-1	7.19 ± 0.34	9.03 ± 0.50	8.33 ± 0.50
	Se-2	34.8 ± 2.2	41.3 ± 5.5	46.6 ± 2.9

* see Table 3; ** Based on 450 µg Se RDA in the EU and UK (Fairweather-Tait et al., 2011).

**Table 7:** Characteristics of the soils.

Characteristic	Sand	Silty Sand	Silt
pH-H_2_O	7.96	6.83	7.34
OM (w/w%)	0.91	1.24	2.12
CaCO_3_ (w/w%)	1.45	0.08	0.20
Total N (w/w%)	0.064	0.092	0.135
NH_4_-N (mg kg^−1^)	1.4	2.3	3.9
NO_3_-N (mg kg^−1^)	4.7	2.3	14.2
AL-K_2_O (mg kg^−1^)	74	174	176
AL-P_2_O_5_ (mg kg^−1^)	131	238	81
CEC (Na meq/100g)	9	17	37
Total Se (mg kg^−1^)	0.076	0.094	0.132
LE Se (mg kg^−1^)	0.009	0.016	0.010
Water soluble Se (mg kg^−1^)	< dl	< dl	< dl
Clay (<0.002 mm, %)	14	23	34
Silt (0.002–0.02 mm, %)	18	30	50
Sand (0.02–2 mm, %)	69	46	16

AL: ammonium-lactate soluble, Total: aqua regia soluble, LE: ammonium-acetate + EDTA soluble, <dl: under detection limit.

**Table 8:** Growth period and irrigation parameters of the vegetables.

Parameter	Green Bean	Cabbage	Potato	Tomato
Growth period	23 May–24 July	17 July–25 September	24 May–17 July	24 May–21 August
Length of growth period (days)	63	71	55	88
Se solution (mL/pot)	7750	19065	10695	27125
Se load in 100 µg L^−1^ treatment (mg/pot)	0.78	1.91	1.07	2.71
Se load in 500 µg L^−1^ treatment (mg/pot)	3.88	9.53	5.35	13.56

**Table 9:** Greenhouse parameters during the growth period of the vegetables.

Parameter	Green Bean	Cabbage	Potato	Tomato
Daytime average temperature (°C)	25.5 ± 3.3	25.5 ± 4	25.6 ± 3.5	26.6 ± 3.3
Nighttime average temperature (°C)	18.3 ± 2.3	18.2 ± 3.4	18.1 ± 2.3	19.1 ± 2.3
Photosynthetically active radiation (W/m^2^)	228.7 ± 106	153.8 ± 43	240 ± 107	214.2 ± 91.6
Air humidity (%)	70.3 ± 8.6	72.2 ± 23	69.7 ± 23.3	69.4 ± 8.1
Soil moisture (% v/v)	24 ± 3	22 ± 6	22 ± 6	22 ± 3

## Data Availability

The datasets analyzed during the current study are available from the corresponding author on reasonable request.

## Supplementary Materials

The following are available online at https://www.mdpi.com/article/10.3390/plants10102086/s1, Table S1: Effect of Se treatment on the fresh and air-dried biomass production of green bean, g·plant^−1^, Table S2: Effect of Se treatment on the fresh and air-dried biomass production of cabbage, g·plant^−1^, Table S3: Effect of Se treatment on the fresh and air-dried biomass production of potato, g·plant^−1^, Table S4: Effect of Se treatment on the fresh and air-dried biomass production of tomato, g·plant^−1^, Table S5: Total and mobilisable element concentrations in the soils (mg·kg^−1^).
