# Supplementary material for: Effect of Se-Enriched Irrigation Water on the Biomass Production and Elemental Composition of Green Bean, Cabbage, Potato and Tomato
Source: Plants (Basel). 2021 Oct 1;10(10):2086. doi: 10.3390/plants10102086 (PMC8537221; doi:10.3390/plants10102086)
Supplement: Supplementary file 1 [file plants-10-02086-s001.zip › plants-1399208-supplementary.pdf]

Effect of Se-enriched irrigation water on the biomass production and elemental composition of green bean, cabbage, potato and tomato

Supplementary tables

Supplementary Table S1. Effect of Se treatment on the fresh and air-dried biomass production of green bean, g·plant^{-1}

Parameter	Se dose	Soil type			Mean
		Sand	Silty sand	Silt	
Root dry weight (g)	Se-0	4.10±1.36b	1.64±0.14a	2.01±0.18a	2.6±1.3a
	Se-1	2.96±0.30ab	1.67±0.01a	2.21±0.53a	2.3±0.6a
	Se-2	2.94±0.40ab	1.72±0.27a	2.44±0.38a	2.4±0.6a
Shoot dry weight (g)	Se-0	16.0±4.1a	12.3±0.4a	13.2±0.6a	13.8±2.7a
	Se-1	12.0±4.2a	11.6±1.5a	10.4±0.9a	11.3±2.4a
	Se-2	13.3±4.2a	13.0±0.6a	13.0±2.7a	13.1±2.5a
Fruit fresh weight (g)	Se-0	86.9±19.9ab	123.0±8.0b	106.1±11.0b	105.4±19.8a
	Se-1	94.8±15.8ab	121.8±6.4b	88.6±5.0ab	101.7±17.7a
	Se-2	66.9±23.9a	119.0±9.9b	89.8±6.2ab	91.9±26.2a
Fruit dry weight (g)	Se-0	8.2±2.6a	13.6±0.5bc	11.3±1.6bc	11.0±2.8a
	Se-1	8.9±2.2ab	14.0±0.7c	9.7±0.2abc	10.9±2.7a
	Se-2	5.9±2.1a	12.9±1.5bc	10.0±1.7abc	9.6±3.4a
Fruit dry matter content (%)	Se-0	9.3±0.9ab	11.1±1.2ab	10.6±0.5ab	10.3±1.1a
	Se-1	9.3±0.9ab	11.5±0.3b	10.9±0.9ab	10.6±1.2a
	Se-2	8.9±0.3a	10.9±0.5ab	11.1±1.2ab	10.3±1.2a

Means±std. dev. Different letters indicate significant differences between any two treatments as well as between the rows in the Mean coloumn for each parameter (Tukey $HSD_{5\%}$)

Supplementary Table S2. Effect of Se treatment on the fresh and air-dried biomass production of cabbage, g·plant^{-1}

Parameter	Se dose	Soil type			Mean
		Sand	Silty sand	Silt	
Root dry weight (g)	Se-0	1.95±0.86a	1.40±0.44a	2.17±0.28a	1.84±0.61a
	Se-1	3.20±0.14a	1.58±0.48a	2.43±0.44a	2.40±0.78ab
	Se-2	2.71±1.52a	3.21±1.17a	2.91±0.56a	2.94±1.02b
Head fresh weight (g)	Se-0	510±226a	476±127a	559±44a	515±136a
	Se-1	446±109a	530±142a	470±39a	482±99a
	Se-2	416±92a	475±22a	473±38a	454±59a
Head dry weight (g)	Se-0	42.9±18.4a	38.9±9.7a	56.0±2.2a	45.9±13.0a
	Se-1	47.9±10.1a	53.1±19.2a	60.4±8.8a	53.8±12.9a
	Se-2	44.4±16.3a	54.7±6.8a	55.0±4.9a	51.4±10.6a
Head dry matter content (%)	Se-0	8.5±0.3ab	8.2±0.3a	10.1±0.5abc	8.9±0.9a
	Se-1	10.8±1.2abc	9.8±1.4abc	12.8±1.2c	11.2±1.7b
	Se-2	10.4±2.0abc	11.6±1.8abc	11.6±0.8bc	11.2±1.5b

Means±std. dev. Different letters indicate significant differences between any two treatments as well as between the rows in the Mean coloumn for each parameter (Tukey $HSD_{5\%}$)

Supplementary Table S3. Effect of Se treatment on the fresh and air-dried biomass production of potato, g·plant^{-1}

Parameter	Se dose	Soil type			Mean
		Sand	Silty sand	Silt	
Root dry weight (g)	Se-0	2.42±0.23a	2.29±0.23a	3.31±1.28a	2.67±0.81a
	Se-1	3.22±0.68a	2.50±1.11a	3.53±0.42a	3.08±0.82a
	Se-2	3.72±0.54a	2.69±0.77a	3.25±0.22a	3.22±0.66a
Shoot dry weight (g)	Se-0	9.71±0.86a	8.98±1.10a	11.00±2.20a	9.89±1.58a
	Se-1	10.76±2.37a	8.34±0.84a	10.37±0.36a	9.82±1.70a
	Se-2	8.39±1.10a	8.86±0.50a	9.21±0.18a	8.82±0.71a
Tuber fresh weight (g)	Se-0	192±7a	191±16a	191±8a	191.2±10a
	Se-1	173±15a	185±1a	186±9a	181.0±11a
	Se-2	165±8a	188±18a	180±14a	177.8±16a
Tuber dry weight (g)	Se-0	38.1±2.0a	36.7±2.8a	36.8±0.3a	37.2±1.9a
	Se-1	34.7±3.3a	35.0±1.7a	37.7±1.9a	35.8±2.5a
	Se-2	30.7±4.0a	38.8±3.1a	35.7±4.3a	35.1±4.8a
Tuber dry matter content (%)	Se-0	19.9±0.3a	19.2±0.5a	19.3±0.7a	19.5±0.6a
	Se-1	20.1±0.4a	18.9±0.9a	20.3±0.0a	19.8±0.8a
	Se-2	18.6±2.4a	20.7±2.3a	20.1±3.8a	19.8±2.7a

Means±std. dev. Different letters indicate significant differences between any two treatments as well as between the rows in the Mean coloumn for each parameter (Tukey $HSD_{5\%}$)

Supplementary Table S4. Effect of Se treatment on the fresh and air-dried biomass production of tomato, g·plant^{-1}

Parameter	Se dose	Soil type			Mean
		Sand	Silty sand	Silt	
Root dry weight (g)	Se-0	2.96±0.16ab	2.52±0.21a	4.16±0.69b	3.21±0.82a
	Se-1	3.02±0.64ab	2.35±0.25a	3.95±0.78b	3.11±0.87a
	Se-2	2.61±0.33a	2.44±0.26a	3.60±0.28ab	2.88±0.60a
Shoot dry weight (g)	Se-0	23.7±3.6a	24.9±1.4a	31.7±6.1ab	26.8±5.2a
	Se-1	27.6±6.3ab	30.7±4.4ab	38.8±7.0b	32.4±7.2b
	Se-2	26.5±2.6ab	27.3±2.9ab	27.9±2.0ab	27.2±2.3ab
Fruit fresh weight (g)	Se-0	372±58a	301±162a	304±120a	326±111a
	Se-1	276±25a	298±131a	209±59a	261±83a
	Se-2	326±34a	309±73a	255±49a	297±57a
Fruit dry weight (g)	Se-0	24.8±2.9a	19.1±10.1a	20.6±6.0a	21.5±6.6a
	Se-1	17.6±1.6a	23.9±10.4a	16.5±5.9a	19.3±7.0a
	Se-2	23.7±2.8a	24.0±3.2a	21.4±3.4a	23.0±3.0a
Fruit dry matter content (%)	Se-0	6.70±0.35abc	6.32±0.39a	7.01±1.03abcd	6.67±0.65a
	Se-1	6.39±0.14ab	8.05±0.06cd	7.74±0.72abcd	7.39±0.85b
	Se-2	7.27±0.15abcd	7.90±0.86bcd	8.44±0.33d	7.87±0.69b

Means±std. dev. Different letters indicate significant differences between any two treatments as well as between the rows in the Mean coloumn for each parameter (Tukey $HSD_{5\%}$)

Supplementary Table S5. Total and mobilisable element concentrations in the soils (mg·kg^{-1})

	Total			Plant available		
	Sand	Silty sand	Silt	Sand	Silty sand	Silt
**Fe**	6574±199	6896±631	7997±1629	42.8±10.2	85.7±11.8	48.0±7.2
**As**	2.88±0.09	3.10±0.39	3.32±0.26	0.139±0.045	0.183±0.104	0.112±0.061
**Se**	0.076±0.012	0.094±0.013	0.132±0.020	0.009±0.009	0.016±0.017	0.010±0.015
**B**	5.23±1.04	9.78±2.73	13.03±4.96	0.740±0.202	0.250±0.194	0.542±0.427
**Mo**	0.110±0.016	0.129±0.012	0.141±0.041	0.016±0.011	0.042±0.020	0.043±0.021
**Cd**	0.129±0.001	0.127±0.014	0.142±0.013	0.071±0.007	0.075±0.018	0.082±0.014
**I**	1.72±0.70	2.26±0.46	3.98±0.66	0.156±0.093	0.160±0.140	0.228±0.096
**Ba**	39.9±3.5	74.3±17.7	139.2±37.5	7.72±0.46	20.4±4.2	30.8±3.5
**Hg**	0.036±0.039	0.051±0.071	0.050±0.054	0.005±0.003	0.004±0.005	0.005±0.004
**Pb**	7.90±0.63	9.67±2.56	11.65±3.40	2.38±0.27	5.07±1.49	4.66±1.00
**V**	12.2±1.2	17.3±5.1	23.9±7.8	0.447±0.118	0.344±0.213	0.495±0.292
**Cr**	10.7±1.6	16.6±5.7	20.5±7.7	0.089±0.025	0.064±0.050	0.050±0.025
**Mn**	246±25	247±75	273±88	94.7±25.3	63.0±42.4	74.1±45.6
**Co**	2.87±0.13	5.20±0.53	6.04±0.36	0.696±0.122	1.73±0.70	2.63±1.2
**Ni**	8.56±0.89	14.2±1.1	17.2±1.1	0.932±0.223	2.27±0.943	3.93±1.82
**Cu**	4.46±0.14	9.29±0.68	10.2±2.0	1.26±0.26	2.96±1.30	3.23±1.56
**Zn**	19.6±1.9	32.6±2.2	33.8±1.6	1.68±0.70	4.78±4.2	2.17±1.6
